# Structural basis for expanded substrate specificities of human long chain acyl-CoA dehydrogenase and related acyl-CoA dehydrogenases

**DOI:** 10.1038/s41598-024-63027-6

**Published:** 2024-06-05

**Authors:** Beena Narayanan, Chuanwu Xia, Ryan McAndrew, Anna L. Shen, Jung-Ja P. Kim

**Affiliations:** 1 Department of Biochemistry, Medical College of Wisconsin, 8701 Watertown Plank Road, Milwaukee, WI 53226 USA; 2 Department of Chemistry and Biochemistry, College of Arts and Sciences, University of North Florida, Jacksonville, FL 32224 USA; 3 McArdle Laboratory for Cancer Research, Department of Oncology, University of Wisconsin-Madison, Madison, WI 53706 USA; 4 Present Address: Molecular Biophysics and Integrated Bioimaging, Lawrence Berkeley National Laboratory, Berkeley, CA 94740 USA

**Keywords:** Biochemistry, Biophysics, Evolution, Structural biology

## Abstract

Crystal structures of human long-chain acyl-CoA dehydrogenase (LCAD) and the catalytically inactive Glu291Gln mutant, have been determined. These structures suggest that LCAD harbors functions beyond its historically defined role in mitochondrial β-oxidation of long and medium-chain fatty acids. LCAD is a homotetramer containing one FAD per 43 kDa subunit with Glu291 as the catalytic base. The substrate binding cavity of LCAD reveals key differences which makes it specific for longer and branched chain substrates. The presence of Pro132 near the start of the E helix leads to helix unwinding that, together with adjacent smaller residues, permits binding of bulky substrates such as 3α, 7α, l2α-trihydroxy-5β-cholestan-26-oyl-CoA. This structural element is also utilized by ACAD11, a eucaryotic ACAD of unknown function, as well as bacterial ACADs known to metabolize sterol substrates. Sequence comparison suggests that ACAD10, another ACAD of unknown function, may also share this substrate specificity. These results suggest that LCAD, ACAD10, ACAD11 constitute a distinct class of eucaryotic acyl CoA dehydrogenases.

## Introduction

**Figure 1:**
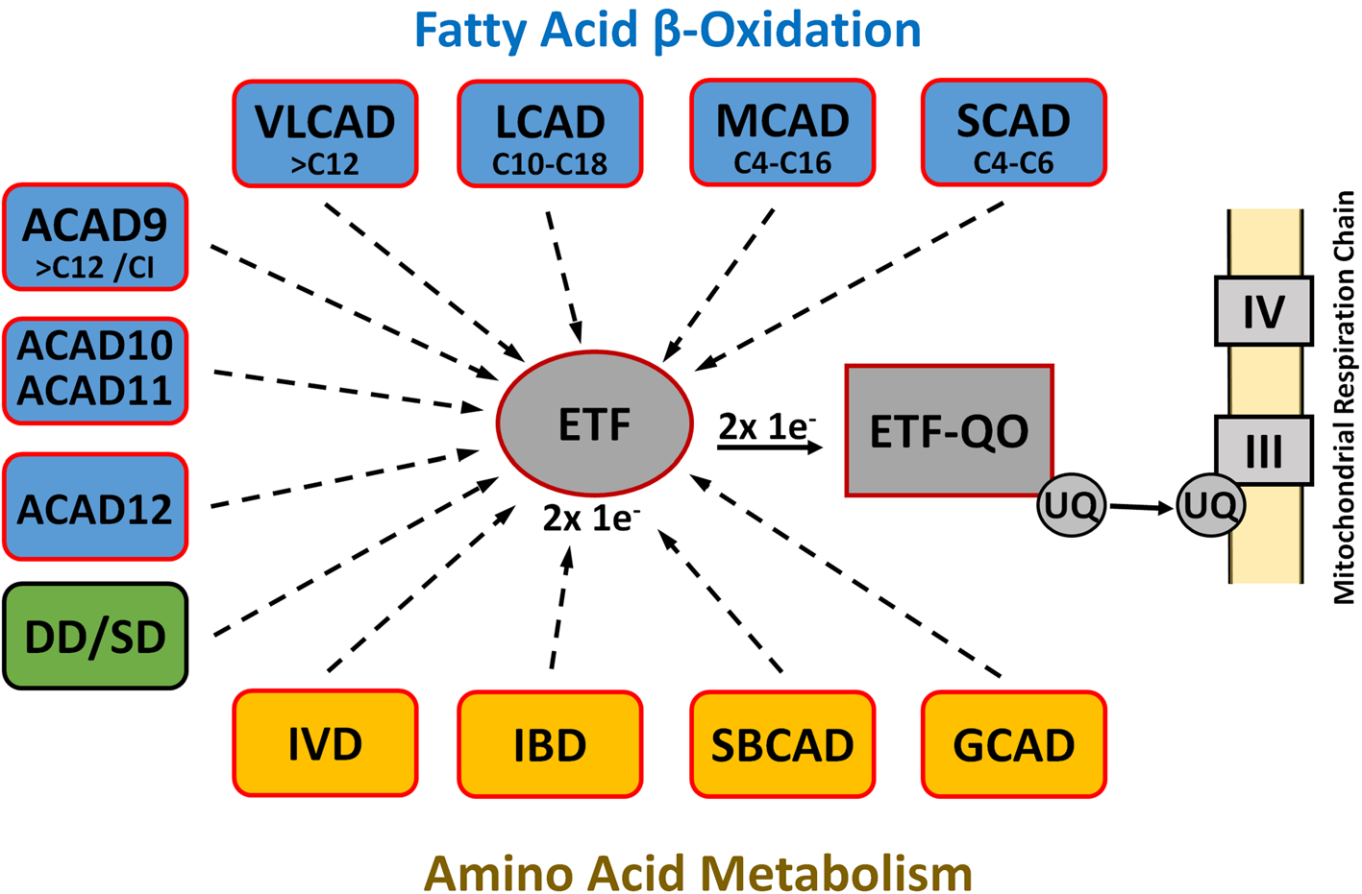
Eucaryotic ACAD family. The eucaryotic ACAD family includes eight ACADs for the metabolism of fatty acids with different but overlapping chain lengths (blue) and four ACADs for amino acid catabolism (yellow). ACAD9 is also involved in the mitochondrial complex 1 assembly. Electron transfer from these dehydrogenases to the main mitochondrial respiratory chain is catalyzed in sequence by electron transfer flavoprotein (ETF) and the membrane-bound ETF-ubiquinone oxidoreductase (ETF-QO), an iron-sulfur flavoprotein. Dimethylglycine dehydrogenase (DD) and sarcosine dehydrogenase (SD) are involved in choline catabolism and interact with ETF, but do not belong to the ACAD family.

Long-chain acyl-CoA dehydrogenase (LCAD), discovered in the 1950s together with its better-known cousin medium-chain acyl-CoA dehydrogenase (MCAD), belongs to an important family of flavoenzymes, acyl-CoA dehydrogenases (ACADs), that catalyze mitochondrial α, β-dehydrogenation of fatty-acyl-CoAs^1^. Five mitochondrial members of this evolutionarily ancient family, with overlapping substrate specificities, have been categorized on the basis of their ability to metabolize fatty acids of varying chain length during mitochondrial fatty acid β-oxidation, although ACAD9 is also essential for mitochondrial complex I assembly^2,3^. In addition, four ACADs (isovaleryl-CoA, isobutyryl-CoA, short branched chain acyl-CoA, and glutaryl-CoA dehydrogenases) involved in amino acid catabolism and sarcosine- and dimethylglycine dehydrogenases, involved in 1-carbon metabolism, have been identified. All of these dehydrogenases, except the dimeric very long chain acyl-CoA dehydrogenase (VLCAD) and ACAD9, are homotetramers containing one noncovalently bound FAD per subunit. Finally, three more ACADs (ACAD10, 11, and 12) have been identified in the human genome and reported to metabolize extremely long (> C20) and branched chain fatty acids^4^ (Fig. 1).

The initial reaction in each cycle of β-oxidation is the dehydrogenation of an acyl-CoA thioester to the corresponding trans-2,3-enoyl-CoA. Catalysis of ACADs is initiated by the abstraction of the *pro-R*-α proton of the thioester substrate and the transfer of a hydride equivalent from the *pro-R*-β-position to the N5 locus of the isoalloxazine ring of the enzyme-bound FAD. Electron transfer from these dehydrogenases to the main mitochondrial respiratory chain is catalyzed in sequence by electron transfer flavoprotein (ETF) and the membrane-bound ETF-ubiquinone oxidoreductase (ETF-QO), an iron-sulfur flavoprotein (Fig. 1).

ACAD deficiencies are among the most common inborn errors of metabolism. The functions of VLCAD, MCAD, and SCAD in oxidation of long, medium, and short-chain acyl-CoAs are well-established, with ACAD-deficient patients typically accumulating intermediate metabolite profiles that reflect the substrate specificity of the deficient enzyme and compensation by other ACADs^5^. LCAD substrate specificity overlaps those of VLCAD and MCAD, and in addition includes unsaturated and branched-chain substrates^6^. LCAD tissue localization and ability to metabolize bulky substrates distinguish LCAD from MCAD and VLCAD^2,6^. No specific metabolic function has been established for this enzyme, though a role in surfactant production has been proposed^7^. Although incidence of MCAD deficiency in the US is 1/12,500, and VLCAD deficiency has a reported incidence between 1/40,000 to 1/120,000, human LCAD deficiency has not been reported. However, studies in laboratory animals have linked LCAD deficiency to heart, liver, and lung pathologies^8–16^ as well as tumor growth^17–19^, but the physiologic function of LCAD remains a mystery.

Here we present the three-dimensional structure of human LCAD, revealing an unusually large substrate-binding cavity not previously reported in eucaryotic ACADs. This structure provides a structural basis for the ability of LCAD to metabolize bulky substrates, including branched-chain fatty acids^6^ and 3α, 7α, l2α-trihydroxy-5β-cholestan-26-oyl-CoA (THC-CoA), an intermediate of the bile acid biosynthetic pathway^2^. Sequence and structural comparisons suggest that this expanded substrate specificity is shared by additional members of the ACAD family, ACAD10 and ACAD11, as well as bacterial steroid-metabolizing ACADs^20^. This expanded substrate specificity calls into question the historical classification of LCAD as an Acyl-CoA dehydrogenase whose primary functions overlap those of MCAD and VLCAD.

## Results and discussion

### Crystallization and overview of the overall fold of the long chain acyl-CoA dehydrogenase

Structures were obtained for wildtype LCAD complexed with and without acetoacetyl-CoA to 2.8 Å and 2.5 Å resolution, respectively. In addition, the structure of the Glu291Gln (E291Q) mutant complexed with lauric acid has been determined to 2.0 Å resolution (see Table 1). In accord with UniProKB, the residue numbering system in this report is based on the 430 amino acid preprotein that includes the 30-residue mitochondrial transport peptide. However, the protein used for our crystallization was the 400 amino acid mature protein without the transport peptide.

**Table 1:** Data collection and refinement statistics.

Data collection	Wt-LCAD	Wt-LCAD	LCAD (E291Q)
PDB code	8W0T	8W0U	8W0Z
Bound ligand	None	Acetoacetyl-CoA	Lauric acid
No. of measured reflections	252,360	309,146	851,736
Unique refs	52,001 (2227)	45,139 (4365)	245,928 (11,015)
Resolution (Å)	50–2.5 (2.59–2.5)	50–2.8 (2.59–2.80)	50–2.0 (2.03–2.0)
Space group	P2_1_2_1_2_1_	P2_1_	P1
Unit cell			
**a** (Å)	101.1	86.0	86.3
**b**	102.1	95.3	94.8
**c**	173.6	118.8	119.2
α (º)	90	90	89.24
β	90	106.52	74.88
γ	90	90	88.35
R_sym_ (last shell)	0.071(0.265)	0.078(0.474)	0.080(0.458)
Completeness (%)	82.8 (47.9)	98.6 (95.8)	96.7(87.1)
I/σi	15.5	11.7	14
Refinement			
Resolution range	30–2.5	50–3.0	50–2.0
R factor (%)	20.0	22.5	21.4
R_free_ (%)	26.2	29.7	25.6
No of water molecules	244	91	1859
Average B factor, Å^2^ (proteins)	49.3	63.2	38.0
Average B factor, Å^2^ (solvent)	36.0	35.9	40.4
Stereochemical ideality (rms deviation)			
Bonds (Å)	0.007	0.006	0.007
Angles (°)	1.3	1.4	1.3
Ramachandran analysis			
Residues in preferred regions (%)	90.7	87.6	95.4
Residues in allowed regions (%)	9.0	12.1	4.3
Residues in disallowed regions (%)	0.3	0.3	0.3

Values in parentheses are for the highest resolution shell.

**Figure 2:**
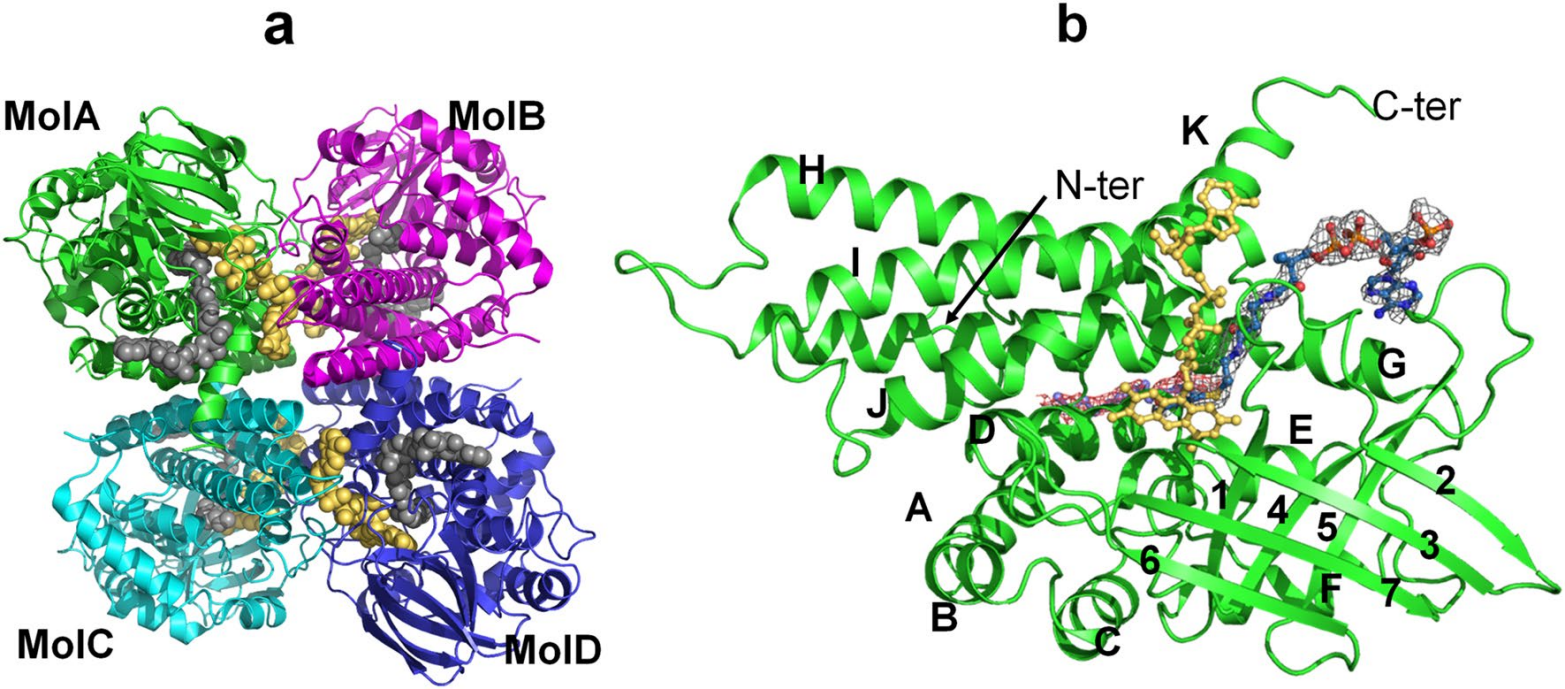
The overall structure of LCAD. (**a**) The LCAD tetramer, or dimer of dimers (MolA:MolB and MolC:MolD dimers), is shown with monomers colored in green (MolA), cyan (MolB), magenta (MolC) and blue (MolD). FAD is shown as yellow balls and C12-CoA as gray balls. (**b**) Representation of LCAD monomer structure with C12-CoA substrate (blue) and cofactor FAD (yellow). The red 2Fo-Fc map at 0.9σ level is derived from the E291Q LCAD crystal structure complexed with lauric acid and the gray 2Fo-Fc map at 1.0σ level is derived from the wildtype LCAD crystal structure complexed with acetoacetyl-CoA. Helices and β-sheets are labelled. Helix A(residues 54–71), B(75–83), C(86–97), D(113–128), E(133–148), F(152–164), β1(166–172), β2(186–192), β3(194–199), β4(211–219), β5(229–237), β6(241–245), β7(258–265), Helix G(279–317), H(327–358), I(361–388), J(396–411), K(414–426).

The overall structure of LCAD is similar to that of the other ACADs whose three-dimensional structures have been determined previously^21–25^. LCAD is a homotetramer and the tetrameric arrangement of the monomers is a dimer of dimers (Fig. 2a). Similar to the other known structures of acyl-CoA dehydrogenases, each LCAD monomer consists of three domains. The ribbon diagram of an LCAD monomer, showing the N-terminal α-helical domain (helices A-F), a β-sheet domain (strands 1–7) and a C-terminal α-helical domain (helices G-K), is presented in Fig. 2b. The first few residues at the N-terminus were not resolved in the wild type and E291Q structures. The chain starts at Glu34 in all four monomers of the mutant LCAD structure. In the wild type structure, the chain starts at Glu34 (monomer A) or Leu36 (monomers B, C, and D). The C-terminal residue Lys430 is observed in all the monomers in the 2.8 Å structure of the wild type protein complexed with acetoacetyl-CoA, but not in the 2.5 Å wild type ligand-free and 2.0 Å (E291Q mutant) structures.

Although no substrates or inhibitors could be co-crystallized with wildtype crystals, the inhibitor acetoacetyl-CoA could be soaked into these crystals. Interestingly, only two monomers (Molecules A and B) of the tetramer have acetoacetyl-CoA bound, presumably due to the crystal packing. Binding of acetoacetyl-CoA to Molecules C and D would result in steric clashes between the adenine ring of FAD in Mol C and loop residues located between Glu151 and His156 of symmetry related Mol A, and between the FAD adenine of Mol D and the loop comprised of Glu105-His106-Leu107 of symmetry related Mol B.

The crystal packing of the LCAD E291Q P1 form is very similar to the crystal packing of the wild type LCAD P2_1_ crystal, except that, in the P1 space group, the adenine ring binding site now has moved slightly away from the symmetry related A and B molecules to avoid steric clashes, transforming the P2_1_ space group of wild type LCAD to the P1 space group of the E291Q mutant crystal. Although we obtained crystals of the LCAD E291Q mutant co-crystallized with substrate lauroyl-CoA (C12-CoA), density for the CoA moiety is not observed, and only weak electron density of the alkyl chain portion is observed, likely due to hydrolysis of C12-CoA during the crystallization process.

As shown in the overlay of LCAD with MCAD and other ACAD structures (Supplementary Fig. S1), the overall fold and topology of LCAD are similar to the other known ACAD structures. The root mean square deviations (RMSDs) between the main-chain atoms of human LCAD and rat SCAD, pig MCAD, and human VLCAD are 1.63 Å, 1.78 Å, and 1.83 Å, respectively.

### Catalytic residue and stereo-specificity of proton abstraction

**Figure 3:**
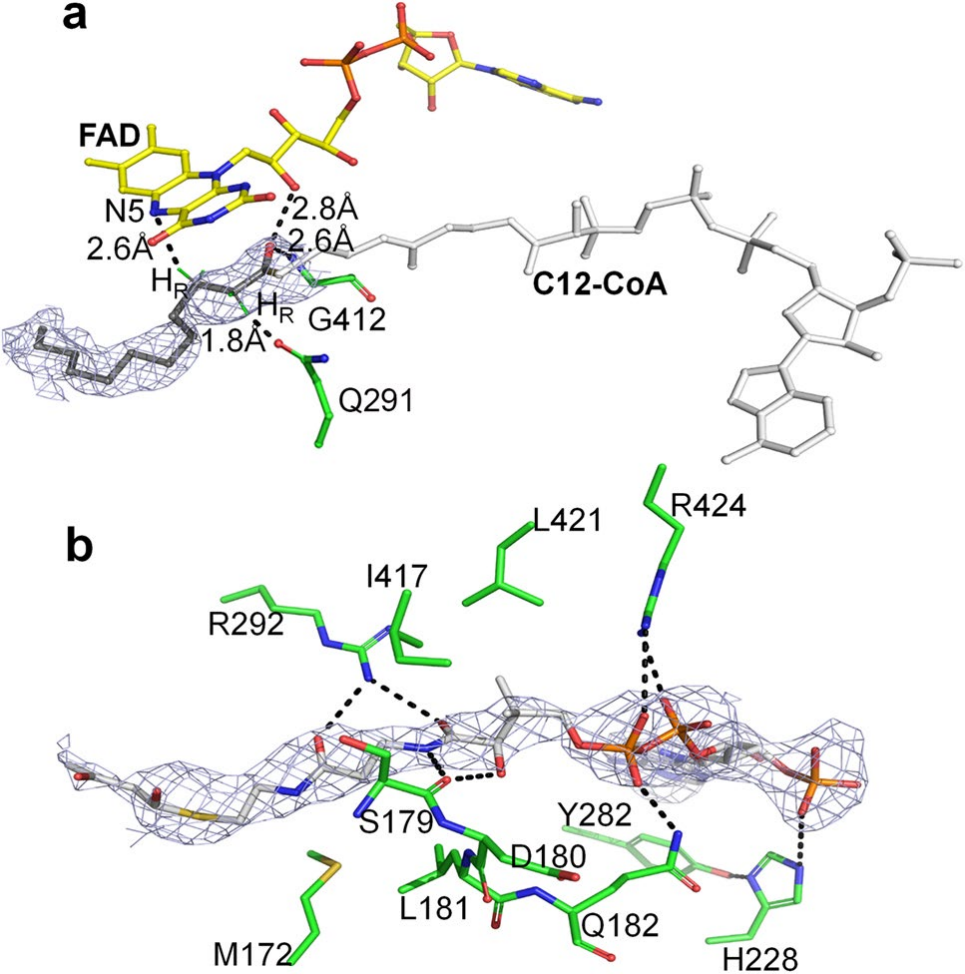
LCAD active site. (**a**) Structure of E291Q LCAD complexed with C12-CoA (PDB code, 8W0Z). Hydrogen bonds are shown as dotted lines with distances indicated. FAD, Gly412, and the catalytic residue (E291Q) are shown as sticks. The C12 *pro-R* hydrogen and Cβ *pro-R* hydrogen are shown in green. The lauroyl moiety is shown as gray sticks and its 2Fo-Fc map at 1.0σ level is shown in gray mesh. The position of the CoA moiety is taken from the structure of the wildtype LCAD complexed with acetoacetyl CoA (PDB code 8W0U) and is shown as white sticks. The distance between Cα of C12-CoA and the carboxylate of E291 is 2.8 Å (inferred from the amide of Gln291 of the mutant structure)) and the distance between Cβ and N5 of FAD is 3.6 Å. The corresponding distances from both α- and β- *pro-R* hydrogens are indicated in the Fig. (1.8 Å and 2.6 Å, respectively). (**b**) Structure of wildtype LCAD complexed with acetoacetyl-CoA, showing hydrogen bonding interactions that stabilize CoA binding (PDB code 8W0U). Hydrogen bonding interactions between LCAD and acetoacetyl-CoA are shown as dotted lines. The 2Fo-Fc map of acetoacetyl-CoA at 1.0σ level is shown in gray mesh. A stereo view is shown in Supplementary Fig. S3.

The structure of LCAD confirms that Glu291 functions as the catalytic base, consistent with previous molecular modeling and site directed mutagenesis studies^26^. The structure of the catalytic residue mutant, E291Q, with the lauroyl moiety of C12-CoA bound in the active site shows that Gln291, extending out from helix G, is located at the active site close to the Cα-Cβ bond of the substrate (Fig. 3a). The distance between the carboxylate of the Glu291 and Cα of the CoA is 2.8 Å, and the distance between the N5 of the FAD and Cβ of the CoA is 3.6 Å. This arrangement of the isoalloxazine ring of the FAD, the Cα and Cβ of the thioester substrate, and the carboxylate of Glu291 is ideally suited for abstraction of the *pro-R* hydrogen as a proton and transfer of the Cβ *pro-R* hydrogen to the N-5 atom of the FAD as a hydride ion^27^, consistent with α-proton abstraction by Glu291. Crystallographic, mutagenesis, and chemical modification studies established Glu405 of MCAD (Glu376 of the mature protein) as the catalytic residue (for review, see Thorpe and Kim^28^). Sequence comparisons among all the members of the acyl-CoA dehydrogenase family show that the location of the catalytic base (Glu405 in MCAD) in the loop between helices J and K is conserved in all but two of the known acyl-CoA dehydrogenases, LCAD and IVD (Supplementary Figure S2). The crystal structure of human IVD complexed with CoA persulfide confirmed its catalytic residue as Glu283 (Glu254 of the mature protein)^23^. Although the catalytic base of MCAD, Glu405, is separated by more than 100 residues from those of LCAD and IVD, these residues are topologically conserved in the 3D structures of these proteins, and the *proR-proR* stereospecificity of the α,β- dehydrogenation reaction is maintained.

As in the structures of other acyl-CoA dehydrogenases, the carbonyl oxygen of the fatty acyl-CoA moiety of the substrate makes hydrogen bonds to the 2’-hydroxyl of the FAD ribityl chain (2.8 Å) and to the peptide backbone of (Gly412-N, 2.6 Å) (Fig. 3a). The corresponding distances in human MCAD are 2.8 and 2.9 Å. These interactions are not only responsible for the orientation of the substrate, but they are also crucial for polarization of the substrate and the lowering of the pK_a_ of the Cα proton for abstraction by the catalytic base, Glu291^29,30^.

### Comparison between the ligand-bound and unbound subunits in wild-type LCAD and between wild-type and mutant subunits

Among the known ACAD crystal structures, MCAD^21^, IBD^25^, and VLCAD^31^ are the only ACADs whose structures reported have been determined both with and without bound substrate or inhibitor. These ligand-bound and unbound ACAD structures have shown that, although subtle changes in the conformations of side chains lining the substrate binding cavity are observed, there are no large conformational changes accompanying ligand binding. Likewise, the overall structure of ligand-bound E291Q LCAD is similar to those of both the ligand bound and unliganded wild-type structures.

In all LCAD structures lacking bound CoA derivatives (all eight monomers of mutant LCAD, all four monomers of ligand-free wild type LCAD, and the C and D monomers of the ligand-bound wild type LCAD), residues Ser179 through Gln182 in the vicinity of the CoA moiety of the substrate are relatively disordered in the absence of ligand. His228 and nearby residues Asp180 and Leu181 are highly mobile, judging from their weak, diffused electron densities. The side chain of Tyr282 is rotated away from the conformation of the bound structure by approximately 125 degrees. New hydrogen bonding interactions contributing to significant CoA binding affinity are observed upon ligand binding (Fig. 3b). Arg424, situated at the entrance of the binding pocket, reorients from a position ~ 5.0 Å from the CoA phosphate into the active site, forming a hydrogen bond (3.3 Å) with the phosphate of the CoA. The side chain of His228, which lies within a hydrogen bonding distance (3.0 Å) of the CoA phosphate, moves up and rotates away (Fig. 3b and Supplementary Figure S3). His228-Nε makes an H-bond with the phosphate of CoA. Having rotated approximately 125 degrees from its position in the unbound structure, the hydroxyl group of Tyr282 makes an H-bond with His228-ND1, so that the phenyl ring of Tyr282 stacks with the adenine ring of CoA. Ser179 is highly conserved and Asp180 is invariant across the ACADs examined, although interactions between the side chains of these residues and CoA are not observed. His228 and Tyr282 are not conserved, but charged residues are found at this position in other ACADs, suggesting that a charge interaction (salt-bridge) can replace the hydrogen bonding interaction.

### Structural basis for substrate chain length and branched chain specificity

**Figure 4:**
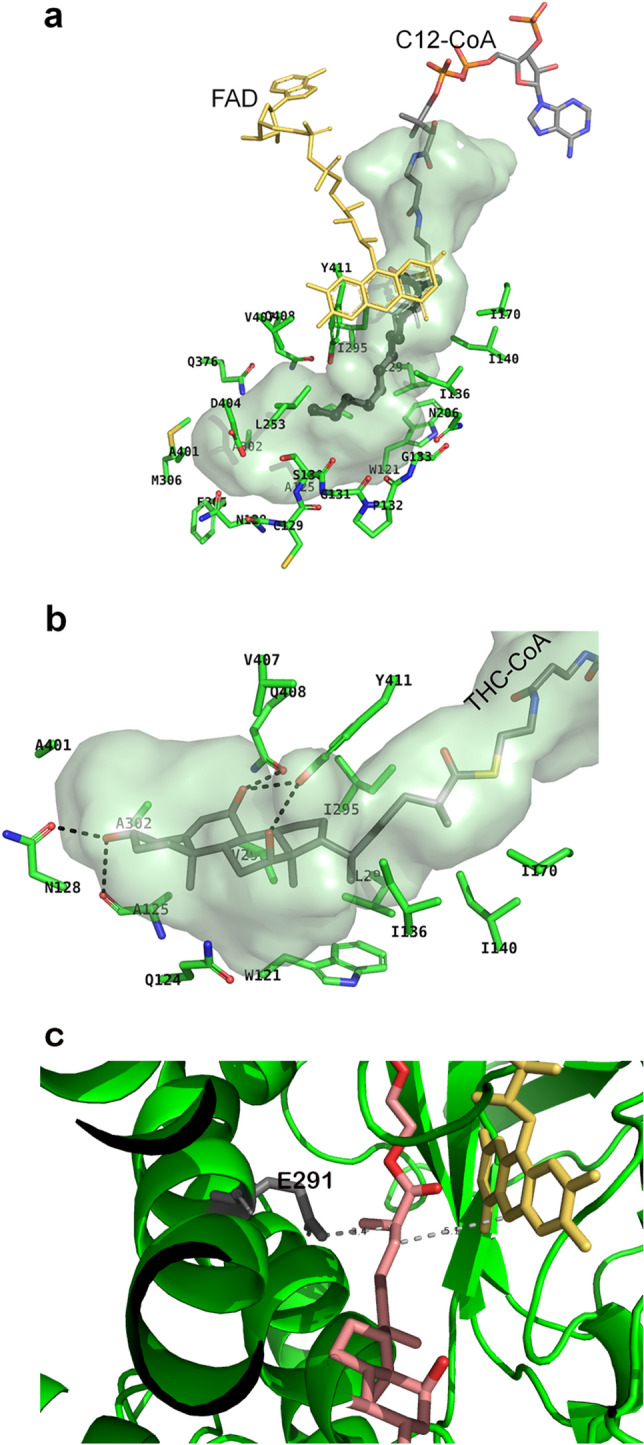
LCAD substrate binding. (**a**) Surface model of LCAD substrate binding site. C12-CoA is shown in sticks with the C12 as thick sticks. FAD is shown as yellow sticks. Amino acid residues lining the substrate binding cavity are: M306, A401, L253, N206, S130, A302, V298, I295, F305, D404, Q376, V407, Y411, W121, I136, I140, I170, L294, Q408, A125; and the P-loop residues (N128, C129, S130, G131,P132, G133), are indicated. (**b**) Surface model of LCAD substrate binding site with THC-CoA. Hydrophobic interactions between THC-CoA and residues A401, V407, A302, A125, Y411, I295, I170, I140, I136, W121, V298, L294 and Q124 are shown. Dotted lines indicate hydrogen bonding interactions between THC-CoA and the side chains of Asn128 and Tyr411 and carbonyl groups of Gln408 and Ala95. (**c**) THC-CoA positioning relative to the catalytic Glu291 and FAD. Glu291 is shown as gray sticks and FAD as gold sticks. THC-CoA is shown as red and salmon sticks. Dotted lines indicate distances between LCAD E291 and Cα of THC-CoA (3.4 A) and between Cβ of THC-CoA and N5 of FAD (5.1 A).

LCAD is active toward substrates with chain length ranging from C10 to C18-CoA with C12- and C14-CoA as the optimum substrates^32^. Figure 4a shows the largely hydrophobic amino acid residues that line the fatty acid binding pocket of human LCAD. The LCAD binding cavity is deeper and wider compared to those of other mammalian ACADs, accounting for the lack of specific interactions between the fatty acyl chain of C12-CoA (bound substrate) and cavity-forming residues. In all eight monomers of E291Q LCAD, electron densities for the substrate fatty-acyl portion are weak and interactions with cavity-forming hydrophobic residues are not seen. This lack of specific interactions may account for the decreased activity of LCAD, in general, compared with MCAD and SCAD^2^.

### Structural basis for metabolism of THC-CoA by LCAD

**Figure 5:**
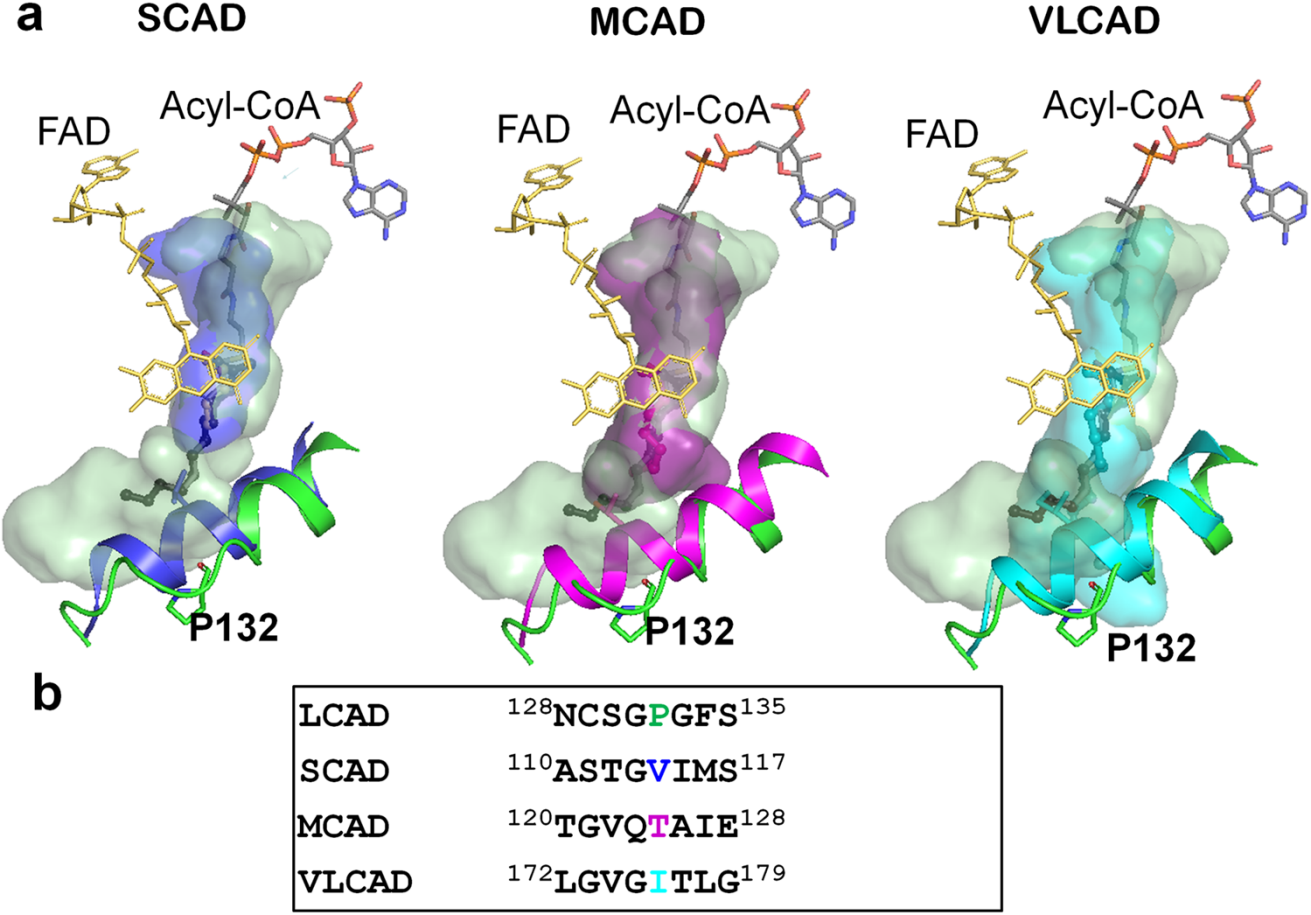
Unwinding of the E helix is the basis for the expanded substrate cavity. (**a**) Overlays of the LCAD substrate binding cavity with those of SCAD (blue), MCAD (magenta), and VLCAD (cyan) are shown along with overlays of helix E (LCAD residues Phe134 to His148). The LCAD E helix is shown in green. (**b**) Sequence alignment in the vicinity of LCAD Pro132. Note that due to Pro132, the LCAD E-helix is shorter than those of SCAD, MCAD, and VLCAD.

Consistent with previous reports of metabolism of THC-CoA by LCAD^2^, inspection of the active site cavity reveals that it is considerably larger than required for C12-CoA (Fig. 4a) and large enough to accommodate THC-CoA. Figure 4b shows a model of THC-CoA bound to the LCAD active site pocket. Hydrogen bonding interactions between THC-CoA and residues Ala125, Asn128, Gln408, and Tyr411 are shown. Figure 4c shows that THC-CoA is positioned to permit proton abstraction from Cα by Glu291 and hydride transfer from Cβ to FAD. The structural basis for the unique substrate binding cavity can be seen in the long E helix, where Pro132 is located in a position to cause unwinding of about two turns of the E helix, thus creating a large cavity. Figure 5a shows overlays of the LCAD substrate binding cavity and E-helix with those of SCAD, MCAD, and VLCAD, showing that unwinding of the helix due to Pro132 allows expansion of the cavity to accommodate longer-chain fatty acids and the steroid moiety of THC-CoA. In addition, small residues in the immediate vicinity of Pro132 (Cys129, Ser130, Gly131, and Gly133) contribute to widening of the cavity (Fig. 5b).

The large active site cavity of LCAD, capable of accommodating bulky substrates including steroid derivatives, suggests that, when smaller substrates are bound, the cavity contains ample water with dissolved air (O_2_) that could contribute to oxidase activity with straight-chain fatty acyl CoAs described by Zhang et al.^33^.

### Stabilization of LCAD tetramer

**Figure 6:**
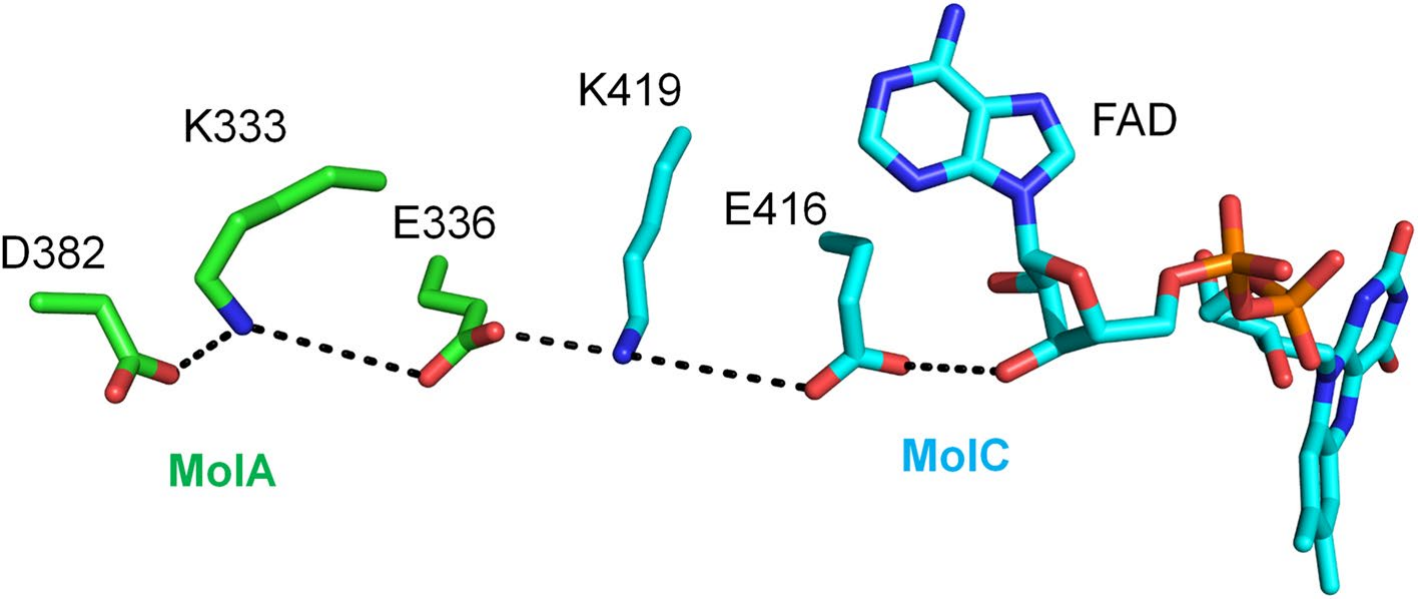
Stabilization of LCAD tetramer conformation by K333. D382/K333/E336 of one LCAD monomer (MolA, green carbon atoms) and K419/E416 of another monomer (MolC, cyan atoms) form a salt bridge chain that stabilizes the tetramer conformation. Dotted lines indicate these salt bridges and a H-bond between E416 and the 3’-OH of the FAD adenosyl-ribose ring stabilizing the FAD binding.

As with other ACADs, FAD is bound at the interface of the LCAD dimer and binding is dependent upon stability of the tetrameric structure. The Lys333Gln (K333Q) polymorphism has been reported to be associated with decreased enzyme activity, protein stability, and FAD content^34^. This residue is located in a chain of salt bridges between two monomers that can stabilize the tetramer conformation (Fig. 6). Disruption of this salt bridge link may destabilize the tetramer and account for both the decreased FAD content and enzyme activity observed in the K333Q form of human LCAD.

### Interaction between ETF and long-chain acyl-CoA dehydrogenase

In the mammalian mitochondrial matrix, electron transfer flavoprotein (ETF) links the activity of at least nine different acyl-CoA dehydrogenases to the respiratory chain by accepting and subsequently transferring electrons to the membrane bound ETF-ubiquinone oxidoreductase (ETF-QO) (Fig. 1).

An overlay of the LCAD:ETF (Supplementary Fig. S4) complex structure based on the previously solved structure of MCAD:ETF^35^ showed that the side chain of βLeu195 of ETF nestles into a hydrophobic pocket formed by LCAD residues Phe60, Gly97, Leu98, Val101, Ile110, Gly112 and Val120, corresponding to the hydrophobic pocket formed by residues Phe 52, Gly89, Leu90, Thr93, Leu102, Leu104 and Ile112, in the MCAD:ETF complex (Supplementary Fig. S2)^35^. Although the side chain of Phe60 of LCAD is oriented ~ 180° away from that seen in MCAD:ETF complex, it forms close contacts with βLeu195 of ETF. The ionic interaction between Glu212 of MCAD and αArg249 of ETF plays a key role to stabilize electron transfer competent state^35^. Leu248 is found at the corresponding position in LCAD; however, the nearby residue Glu260 is in a position to form the salt bridge with ETF αArg249 (Supplementary Fig. S2).

Circadian regulation of LCAD and MCAD has been demonstrated and associated with SIRT3-mediated deacetylation^36^. LCAD Lys318 and Lys322 have been identified as targets of SIRT3 and are also conserved in MCAD and ACAD9 (Supplementary Fig. S2) as SIRT3 targets^37^. The location of these residues, on the surface of the protein and in a position to interact with ETF^35^, provides a structural basis for circadian regulation of these enzymes via SIRT3-mediated deacetylation. Although Lys42 has also been identified as a SIRT3 target^38^, mutation of this residue did not inhibit SIRT3-mediated deacetylation^37^ and its role in regulation of LCAD activity remains to be established. This residue is conserved in LCAD and ACAD9, but not MCAD (Supplementary Fig. S2).

### Expanded binding site cavities in mammalian and bacterial ACADs

**Figure 7:**
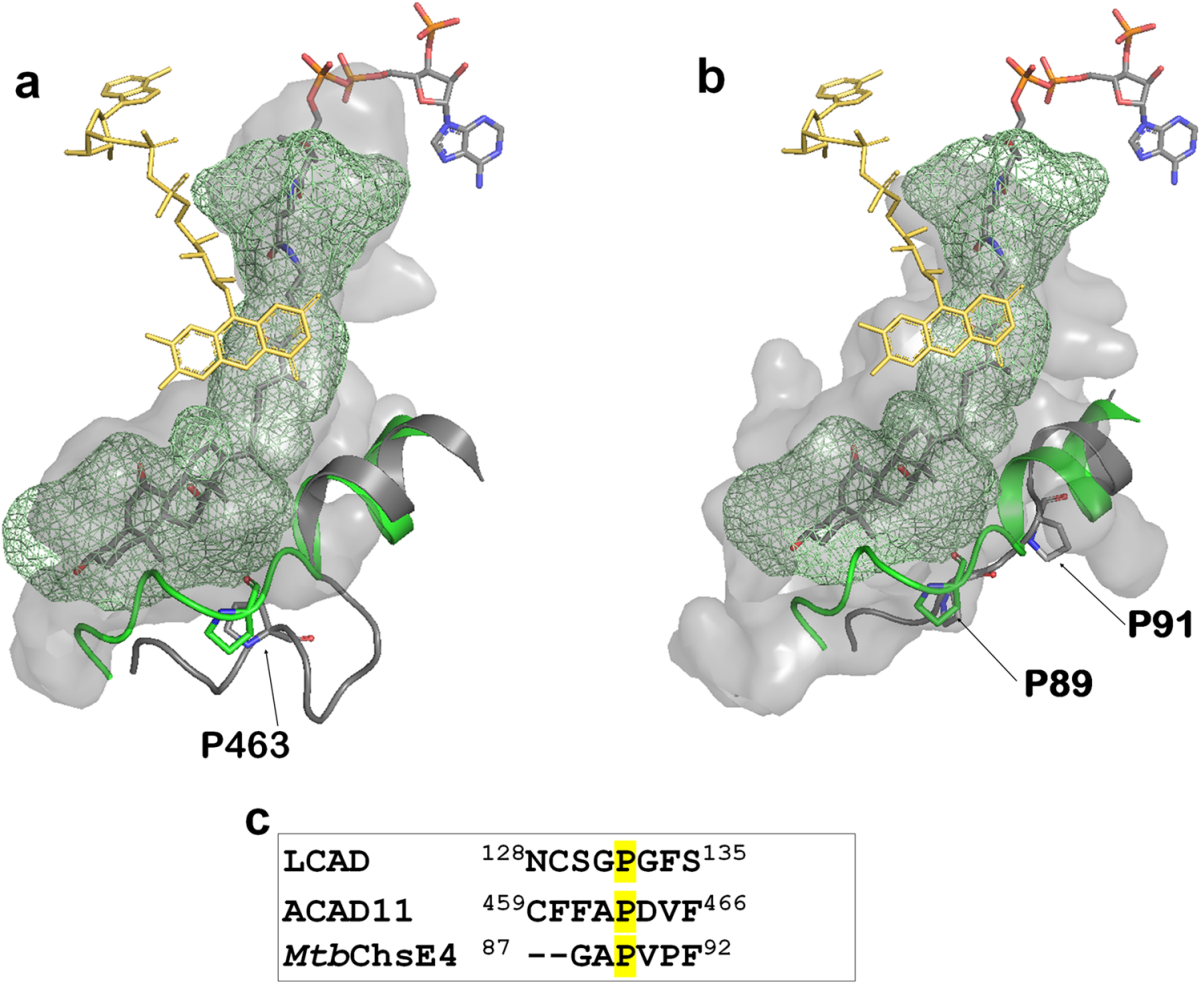
Similarity/conservation of LCAD, ACAD11 and *Mtb*ChsE4 substrate binding cavities. (**a**) LCAD substrate binding cavity (green mesh) overlaid onto ACAD11(pdb code, 2wbi) substrate binding cavity (surface model) with the substrate THC-CoA. The LCAD E-helix with Pro132 is shown in green and the corresponding ACAD11 helix with Pro463 is shown in gray. (**b**) LCAD substrate binding cavity (green mesh) overlaid onto *Mtb*ChsE4 (pdb code, 4X08) substrate binding cavity (surface model) with the substrate THC-CoA. The LCAD E-helix with Pro132 is shown in green and the corresponding *Mtb*ChsE4 helix with Pro89 and Pro91 is shown in gray. (**c**) Sequence alignment in the vicinity of LCAD Pro132.

Identification of the role of Pro132 in partial unwinding of the E-helix prompted a search for other ACADs that might share this expanded substrate-binding cavity. Alignment of LCAD with ACAD10, ACAD11, and ACAD12 reveals the presence of a proline at residues 782 and 463, respectively, of ACAD10 and ACAD11 (Supplementary Figure S2). Examination of ACAD11 structure (PDB code:2wbi) reveals unwinding of the helix at Pro463, permitting binding of a bulky substrate such as cholesterol (Fig. 7a). In addition, the ACAD11 binding site is further expanded to accommodate longer-chain substrates, consistent with the reported ability of ACAD11 to oxidize substrates as long as C24^4^. No structure of ACAD10 has been determined, but sequence similarity to LCAD and ACAD11 suggests the presence of a large substrate binding cavity. Although metabolism of 2-methyl C15-CoA by ACAD10 has been demonstrated, metabolism of THC-CoA has not been tested^4^. ACAD12 does not contain a proline residue at this position, suggesting that it does not share this large substrate binding cavity.

Bacteria, notably *Mycobacterium tuberculosis*, are known to utilize steroids as carbon sources^20,39^. Thomas et al. have identified a unique α_2_β_2_ heterotetrameric ACAD, encoded by separate genes and distinct from the homotetrameric eucaryotic ACADs^40^, that carry out β-oxidation of the cholesterol side chain. The crystal structures of two of these α_2_β_2_ ACADs, ChsE4-ChsE5 (FadE26-FadE27) and ChsE1-ChsE2 (FadE28-FadE29)^20^, have been determined, showing that the α subunit binds the FAD isoalloazine, contains the catalytic Glu residue, and substrate binding site; while the β subunit interacts with the ADP-ribose moiety of FAD. Comparison of the structure of ChsE4 with that of LCAD demonstrates the large active site cavity, showing helix unwinding at Pro89 of ChsE4, corresponding to Pro132 of LCAD*.* Although Pro89 in ChsE4 is conserved as in LCAD and ACAD11, there is another proline (Pro91) in ChsE4. Because of this additional proline, the Pro-loop in ChsE4 is longer than those in LCAD and ACAD11, resulting in a slightly larger cavity in the structure of ChsE4, as shown in Fig. 7b (see the gray surface model).

### Evolutionary origins of LCAD

LCAD is historically classified as a member of the acyl-CoA dehydrogenase family responsible for mitochondrial β-oxidation of fatty acyl-CoA thioesters, that includes SCAD, MCAD, LCAD, VLCAD, and ACAD9. Phylogenetic analysis has shown that individual eucaryotic ACADs bear greater sequence similarity to bacterial ACADs than to other eucaryotic ACADs, suggesting that clades containing these five eucaryotic ACADs diverged well before the common ancestor of Archaea, Bacteria, and Eucarya^41^. Although LCAD shares limited sequence identity (24–34%) with other eucaryotic ACADs, BLAST searching of bacterial and archaeal genomes revealed that an ACAD from *Sneathiella chungangensis* (WP_161340043.1) displayed the greatest homology to human LCAD, with 59% amino acid sequence identity, while *M. tuberculosis* contains ACADs with as high as 52% sequence identity. Examination of the aligned sequences of human and *M. tuberculosis* ACADs (Supplementary Fig. S2) reveals a complex evolutionary pattern with preservation of functional residues. The catalytic LCAD Glu291 is conserved in IVD and the *M. tuberculosis* ACADs. Glu405 of MCAD is conserved in MCAD, VLCAD, SCAD, and IBD, while ACADs 10, 11, and 12 and *Mtb*4HR3 contain aspartate at the position corresponding to MCAD Glu405. Residues forming the hydrophobic ETF binding pocket are largely conserved in all the ACADs. The salt bridge with ETF αArg249 is preserved in human ACADs, with conservation of LCAD Glu260 in LCAD, IVD, and ACADs 10–12 and conservation of MCAD Glu241 in MCAD, SCAD, and IBD. Interestingly, Glu is found at both positions in VLCAD and ACAD9.

Pro132, responsible for helix unwinding and expansion of the substrate binding cavity, is conserved in LCAD and the four *M. tuberculosis* ACADs, including *Mtb*ChsE4 and *Mtb*ChsE2 that are known to metabolize cholesterol (Fig. 7b and Supplementary Figure S2, and Yang et al.^20^). Although substrate specificities of ACAD10, ACAD11, *Mtb*4HR3_A, and *Mtb*CNF74574 are unknown, the presence of the proline residue in the E helix suggests that they may also metabolize sterol substrates.

### Physiological functions of LCAD

All reported human cases of long chain fatty acid dehydrogenase deficiency have been linked to VLCAD mutations and no human case of LCAD deficiency has been reported. However, although LCAD substrate specificity overlaps with those of VLCAD and MCAD, the minor allele frequency of the Glu291Lys (E291K) polymorphism, resulting in elimination of the catalytic base, is very low (1.10e-5, https://gnomad.broadinstitute.org/variant/2-211068168-C-T), suggesting an essential function for LCAD that cannot be replaced by VLCAD or MCAD. A noncoding polymorphism in the human *ACADL* gene has been associated with alterations in serum 2,6-dimethylheptanoic acid levels^42,43^. The ability of LCAD to accommodate bulky substrates such as branched chain fatty acids and sterol substrates is not shared by SCAD, MCAD, VLCAD, or ACAD9, suggesting that metabolism of one of these compounds is an essential function of LCAD.

Fasting-induced hypoketotic hypoglycemia and cardiac hypertrophy seen in both LCAD- and VLCAD-null mice are similar to symptoms seen in human VLCAD deficiency. LCAD knockout mice also exhibit fasting-induced hepatosteatosis and cardiac dysfunction in association with altered branched-chain amino acid metabolism, decreased anaplerosis and activation of the integrated stress response^12,15^. Although VLCAD mRNA levels are 85 times that of LCAD in human heart and skeletal muscle^2^, suggesting that VLCAD is primarily responsible for long chain fatty acid metabolism in these tissues, LCAD has been identified as a modulator of cardiac remodeling in a mouse model of stress-induced hypertrophy^44^. Circadian regulation of LCAD via Sirt3-mediated deacetylation suggests a role in energy production in response to feeding and fasting cycles^36–38^ and in vitro studies have implicated LCAD as a tumor suppressor in human cancer^17–19,45,46^.

In human lung, LCAD is localized to ATII cells responsible for synthesis and secretion of pulmonary surfactant, a mixture of phospholipids, cholesterol, and surfactant proteins, and LCAD knockout mice exhibit surfactant dysfunction, decreased lung compliance and increased susceptibility to influenza infection^7^. Although regulation of cholesterol content is essential for surfactant structure, mechanisms of cholesterol synthesis, uptake and clearance are not well understood^47^. Single cell transcriptomic analysis of pulmonary fibrosis has identified a cholesterol metabolic process and downregulation of LCAD^48^, raising the possibility that sterol metabolism may be a function of LCAD in alveolar ATII cells.

In conclusion, we present structural evidence showing that LCAD is a prototype of a distinct class of eucaryotic ACADs that has evolved an enlarged substrate-binding cavity suitable for β-oxidation of bulky substrates including branched chain fatty acyl-CoAs and sterol derivatives. This structure, where the presence of proline in the E helix leads to formation of a large active-site cavity, has also been found in steroid-metabolizing ACADs in bacteria and ACAD11 in eukaryotes. Sequence similarity suggests that substrate binding site of ACAD10 may share this structure. The expanded substrate specificity of LCAD raises the possibility of multiple functions for this enzyme in normal physiology, as well as a mechanism for metabolic switching during pathological states such as cardiomyopathy and tumorigenesis.

## Materials and methods

### Expression and purification of human wild-type and mutant LCAD

The enzyme was expressed and purified as previously described^26,32^. Briefly, the expression plasmid pET11a-LCAD was transformed into *Escherichia coli* strain BL21(DE3). The cells were grown in 2YT medium with 50 μg ampicillin/ml and 34 μg chloramphenicol/ml at 28 °C to an optical density (A_600_) of 0.9–1.0. Expression of LCAD was induced by addition of isopropyl-thio-β-d-galactoside to a final concentration of 0.1 mM and grown at 30 °C for 17 h. The cells were then harvested and sonicated in a buffer containing 20 mM potassium phosphate buffer, pH 7.0, and 10% glycerol. Broken cells were centrifuged, and the supernatant loaded on a DEAE column and eluted with a 20-320 mM phosphate gradient, pH 7.0. Fractions containing LCAD were pooled based on activity and further purified by hydroxyapatite chromatography using 20-350 mM phosphate gradient, pH 7.0. The protein concentrations were determined based on 447 nm extinction coefficient of 13.3 mM^−1^ cm^−1^ per flavin and the Bradford protein assay^49^.

### LCAD activity assay

LCAD activity was determined using the ferricenium assay with ferricenium as the terminal electron acceptor^50^. The reaction was initiated by addition of C12-CoA substrate. Reactions were monitored on a Shimadzu UV-160 spectrophotometer. The activity of the LCAD protein used for crystallization was 15.3 (± 1) units/mg protein. (1unit = 1 µmol of ferricenium reduced/min, using C12-CoA as the substrate.)

### Crystallization, data collection, and structure determination

All crystals were grown using the hanging drop vapor diffusion method at 19 °C. The initial wild type LCAD crystals were obtained by mixing equal volumes of protein solution (18 mg/ml; 20 mM Tris–Cl, pH 8.5 & 100 mM NaCl) and a reservoir solution (250 mM Tris–Cl, pH 8.2, 17% (w/v) PEG monomethyl ether 5000, 100 mM NaCl, 1 mM MgCl_2_). The crystals were flash frozen in liquid nitrogen after soaking in cryo-solution containing the precipitating solution with 10% glycerol. The wild type data were recorded at –170 °C to 2.5 Å using an in-house X-ray instrument of R-AXIS IV^++^ detector system with a Micromax 007 generator. The first wild type LCAD crystal was indexed in the space group P2_1_2_1_2_1_. Assuming one tetramer per asymmetric unit the calculated Vm is 2.5Å^3^/Da with 50.1% solvent content. Data reduction and scaling were performed using HKL2000^51^. The initial LCAD structure (wild type apo structure) was solved by the Phaser molecular replacement using pig MCAD tetramer structure (pdb code: 3MDE) without any substrates and cofactors. The initial solution has an RFZ of 5.6, TFZ of 10.6 and LLG of 125. The strong electron density map in the FAD position indicated the correctness of the solution. A round of energy minimization consisting of rigid body, positional and simulated annealing refinement using CNS^52^ reduced the R-factor to 45.2%. Manual rebuilding of the model was performed at this stage. The correct LCAD residues were inserted when the electron density was observed in the difference Fourier maps. After several rounds of iterative cycles of energy minimization followed by manual model building the entire LCAD sequence was correctly modeled using the COOT program^53^. The final working R-factor was refined to 20.0% with R-free of 26.2%.

When the wild type LCAD protein was crystallized in the same condition with 0.1% detergent β-octyl-β-d-glucoside in both protein solution and precipitant solution, the wild type crystals were grown in P2_1_ space group with one tetramer in one asymmetric unit. Under these conditions, though no substrates or inhibitors could be co-crystallized with wildtype LCAD, the inhibitor acetoacetyl-CoA can be soaked into the crystals. Using the wild type apo LCAD structure, Phaser resulted in a solution with RFZ of 9.7, TFZ of 10.2 and LLG of 4248. The structure of wild type LCAD complexed with acetoacetyl-CoA was refined to a final R-working of 22.5% and R-free of 29.7% at resolution of 2.8 Å.

The best crystals were obtained when a substrate C12-CoA was co-crystallized with an inactive LCAD mutant, E291Q, in the same condition as above, i.e. mixing equal volumes of protein solution (18 mg/ml; 20 mM Tris–Cl, pH 8.5 and 100 mM NaCl, 0.1% β-octyl-β-d-glucoside) and a reservoir solution [250 mM Tris–Cl, pH 8.2, 17% (w/v) PEG monomethyl ether 5000, 100 mM NaCl, 1 mM MgCl_2_, 0.1% β-octyl-β-d-glucoside]. The mutant crystal data were collected in SBC 19ID beamline at the Advanced Photon Source, Argonne National Laboratory and indexed in P1 space group with a resolution of 2.0 Å. There are two tetramers in one asymmetric unit. The two tetramer solutions in phaser molecular replacement were, RFZ = 7.9 TFZ = 10.6, LLG = 551, and RFZ = 7.9, TFZ = 8.5, LLG = 528. The final crystal structure has a Rwork of 21.4% and Rfree of 25.6%. Although the mutant was co-crystallized with substrate C12-CoA, the density for the CoA part was not observed, and only weak electron density of the alkyl chain portion was observed, likely due to the hydrolysis of C12-CoA during and or after the crystallization process. All eight monomers contain similar electron density for the fatty acid portion in the substrate-binding pocket, indicating the hydrolysis of C12-CoA was likely occurred after the C12-CoA crystals were formed.

The final data collection and refinement statistics are summarized in Table 1 and the structural data have been deposited into Protein Data Bank with pdb codes of 8W0T, 8W0U, and 8W0Z.

For modelling of *3α, 7α, l2α-trihydroxy-5β-cholestan-2β-oyl-CoA* (THC-CoA) into the active site, the 2D chemical structure of THC-CoA was obtained using https://pubchem.ncbi.nlm.nih.gov/compound/122312#section=3D-Conformer. PDB coordinates along with its topology file were generated using PRODRG program in CCP4^54^. The THC-CoA molecule was then manually docked into the LCAD active site, and the entire complex was refined using Rigid Body and Minimization Refinement in the CNS program^52^.

### Supplementary Information

Supplementary Figures.

## Data Availability

The structural data have been deposited into Protein Data Bank with pdb codes of 8W0T, 8W0U, and 8W0Z.
