# Supplementary material for: Structural basis for expanded substrate specificities of human long chain acyl-CoA dehydrogenase and related acyl-CoA dehydrogenases
Source: Sci Rep. 2024 Jun 5;14:12976. doi: 10.1038/s41598-024-63027-6 (PMC11153573; doi:10.1038/s41598-024-63027-6)

Supplementary Information for

Structural Basis for Expanded Substrate Specificities of Human Long Chain Acyl-CoA Dehydrogenase and Related Acyl- CoA Dehydrogenases.

Beena Narayanan, Chuanwu Xia, Ryan McAndrew, Anna L. Shen, and Jung-Ja P. Kim

Supplementary Figure Legends.

Figure S1. ACAD structural comparison. Overlay of human LCAD structure (Mol A, green) with those of rat SCAD (1JQI), shown in blue; pig MCAD (3MDE), shown in magenta; and human VLCAD N-terminal domain (3B96, residues 54-445), shown in cyan. C12-CoA is shown as sticks and FAD as gold sticks.

Figure S2. ACAD sequence alignment. Alignment of LCAD protein sequence with other eucaryotic ACADs and related M. tuberculosis ACADs. Alignments were carried out using COBALT (Papadopoulos JS and Agarwala R (2007) COBALT: constraint-based alignment tool for multiple protein sequences, Bioinformatics 23:1073-79). Residues corresponding to LCAD residues 127-139 were manually aligned according to structural comparisons of LCAD, SCAD, MCAD, VLCAD, MtbChsE4, and ACAD11. Percent identity of each sequence to LCAD is indicated in parentheses. Conserved residues are shown in red. Positions where <50% of the sequences contain gaps are shown in gray lower case. Positions where >50% of the sequences contain gaps are shown in gray upper case. The catalytic Glu or Asp residues are highlighted in green. The position of the conserved LCAD Pro132 is highlighted in yellow. Putative LCAD lysine residues deacetylated by SIRT3 are shown in bold. Gray bars highlight residues homologous to MCAD ETF-interacting residues. Glu residues interacting with Arg249 of ETF are indicated by bold underline. Accession numbers are LCAD, NP_001599.1; *Mtb*CNF74574, CNF74574; IVD, NP_002216.3; SCAD, AAD00552.1; IBD, NP_055199.1; MCAD, NP_001120800.1; ACAD9, NP_054768; VLCAD, BAA29057.1;*Mtb*4HR3_A, 4HR3_A; *Mtb*ChsE4, I6YCA3; *Mtb*ChsE2, P71858.2; ACAD10, NP_001130010; ACAD11, NP_115545; Acad12, NP_848914.2.

Figure S3. A stereo figure for the electron density of acetoacetyl-CoA bound to the wild-type LCAD, showing hydrogen bonding interactions that stabilize CoA binding. Hydrogen bonding interactions between LCAD and acetoacetyl CoA are shown as dotted lines. The 2Fo-Fc map of acetoacetyl-CoA at 1.0δ level is shown in gray mesh.

Figure S4. LCAD-ETF complex. LCAD monomers are shown in green, magenta, cyan and blue riboons. The ETF alpha subunit is shown in gold and the beta subunit in gray. FAD is shown as gold sticks. The arrows point to ETF βLeu195, shown as gray sticks. The view shown in (a) is rotated 90° along the Y axis to produce the view shown in (b).

Fig. S1

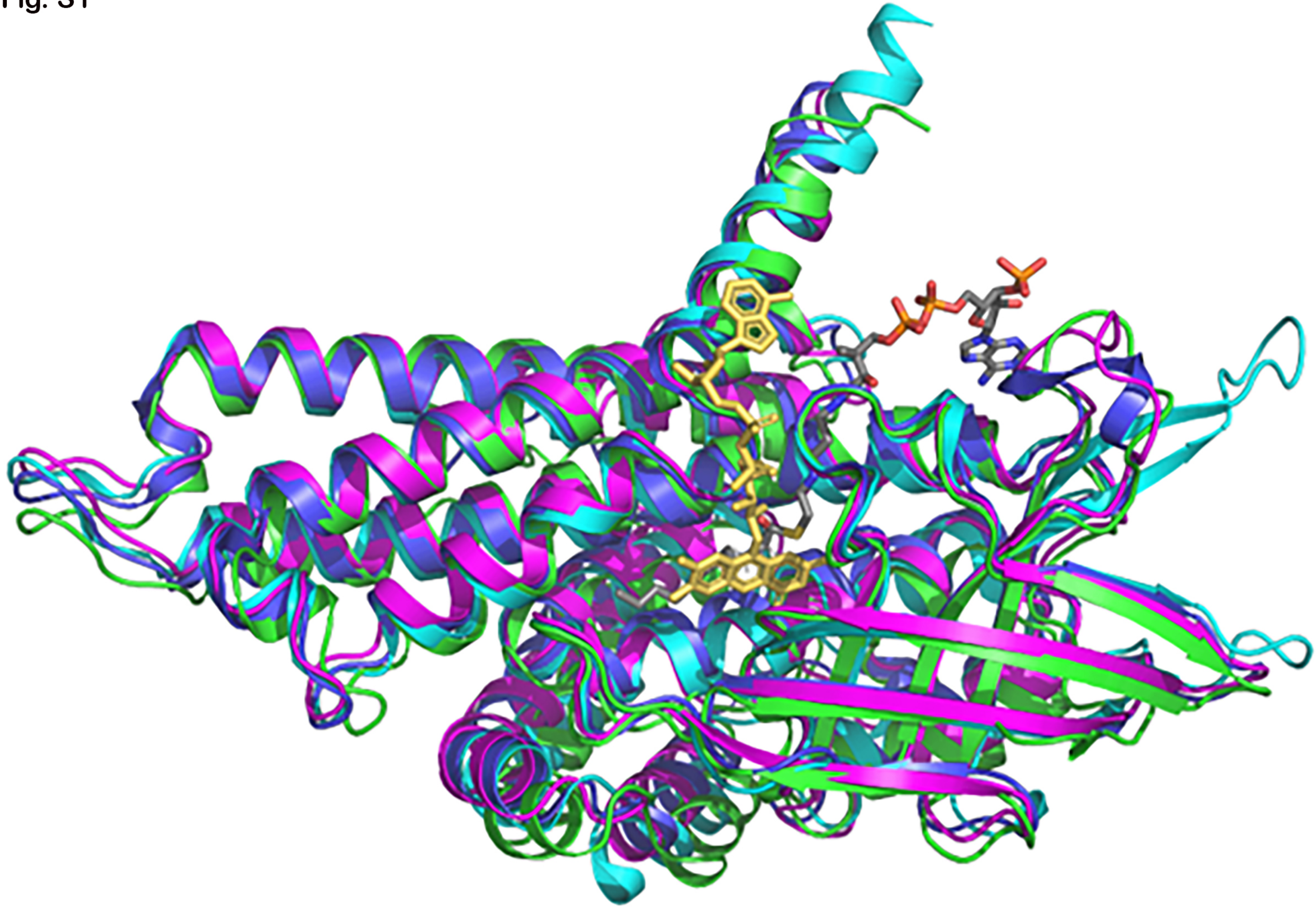

Fig. S2

```
LCAD              1        MAARLLRGSLRVLGG[4]RQLPAA[4]SGGEERLETPsAKKLTDIGIRRIFSPEHDI   FRKSVRKFFQEEVIP  74
MtbCNF74574(52)   1        MG-------------   ------   --------------------RDIYTDEHDA   FRDMVRSFIAKEAAP  27
IVD(34)           1        MATATRLLGWRVASW   RLRPPL[2]FVSQRAHSLL-----PVDDAINGLSEEQRQ   LRQTMAKFLQEHLAP  63
SCAD(33)          1        MAAALLAR----ASG   PARRAL[2]RAWRQLHTIY-----QSVE----LPETHQM   LLQTCRDFAEKELFP  55
IBD(33)           1        MLWSGCRRFGARLG-   ------[3]GGLRVLVQTGhRSLTSCIDPSMGLNEEQKE   FQKVAFDFAAREMAP  62
MCAD(32)          1        MAAGFGRCCR--CSL   QVLRSI[2]FHWRSQHTKAnRQREPGLGFSFEFTEQQKE   FQATARKFAREEIIP  66
ACAD9(31)         1    [ 12]-AARACRGLVVSTAN[4]RTSPPV   RAFAKELFLGkIKKKEVFPFPEVSQDELNE[3]FLGPVEKFFTEEVDS  84
VLCAD(29)         1    [ 42]GAAQLALDKSDSHPS[4]RKKPAK[3]KSFAVGMFKGqLTTDQVFPYPSVLNEEQTQ[4]LVEPVSRFFEEVNDP  119
Mab4HR3_A(28)     1        ---------------   ------   ------------GPGSMDFAP---SARAAE   LIAAVREFIDAEVMP  30
MtbChsE4(27)      1        ---------------   ------   -------------------MRISYTPQQEE   LRRELRSYFATLMTP  26
MtbChsE2(26)      1        ---------------   ------   -------------------MFIDLTPEQRQ   LQAEIRQYFSNLISP  26
ACAD10(26)        1    [652]ARPQSQWCPTGSRSY[4]EASPAH   -----------TSRGGLVISPESLSPPVRE   LYHRLKHFMEQRVYP  711
ACAD11(26)        1    [356]------------RTF[4]---PQI   -----------DTTGQLFVQ----TRKGQE   VLIKVKHFMKQHILP  396
ACAD12(25)        1    [112]AGPRSPRTPKGVRGH[4]AASPSH   -----------EAKGGLVISPEGLSPAVRK   LYEQLVQFIEQKVYP  171

LCAD             75   HHSEWEKA     GEV--S---REVWEKAGKQGLLGVNIAEHLG   GIGGD-LYSAAIVWEEQAYS-N-CS-GPG---FSIHSG  139
MtbCNF74574      28   HHQQWEKD     GIV--S---REVWLAAGRAGLLGIDMPEEYG   GGGNDdYRYYVIFNEELAKA-G-VH-GPG---FAVHND  93
IVD              64   KAQEIDRS     NEFKNL---REFWKQLGNLGVLGITAPVQYG   GSGLG-YLEHVLVMEEISRA-S-GAVGLS---YGAHSN  131
SCAD             56   IAAQVDKE     HLF--P---AAQVKKMGGLGLLAMDVPEELG   GAGLD-YLAYAIAMEEISRGCA-ST-GVI---MSVNNS  121
IBD              63   NMAEWDQK     ELF--P---VDVMRKAAQLGFGGVYIQTDVG   GSGLS-RLDTSVIFEALATG-C-TS-TTA---YISIHN  127
MCAD             67   VAAEYDKT     GEY--P---VPLIRRAWELGLMNTHIPENCG   GLGLG-TFDACLISEELAYGCT-GV-QTA---IE-GNS  131
ACAD9            85   --RKIDQE     GKI--P---DETLEKLKSLGLFGLQVPEEYG   GLGFS-NTMYSRLGEIISM--D-GSITVT---LAAHQA  147
VLCAD           120   --AKNDAL     EMV--E---ETTWQGLKELGAFGLQVPSELG   GVGLC-NTQYARLVEIVGMHDL-GV-GIT---LGAHQS  183
Mab4HR3_A        31   VERAVLAH[13]ELWHVPpelDSLKAKARAAGLWNLFLPDPE-[1]GGGLS-NSEYAPLAEQMGRS-LfAPTVFN---CNAPDS  115
MtbFaDE26        27   ERREALSS[ 2]GEYGVGnvyRETIAQMGRDGWLALGWPKEYG   GQGRS-AMDQLIFTDEAAIA----G-APV---PFLTIN  96
MtbFaDE29        27   DERTEMEK     DRHGPA--yRAVIRRMGRDGRLGVGWPKEFG   GLGFG-PIEQQIFVNEAHRA-D----VPL---PAVTLQ  92
ACAD10          712   AEPELQSH[ 4]ARWSPSpliEDLKEKAKAEGLWNLFLPLEAD[5]GAGLT-NVEYAHLCELMGTS-LyA---PEVCNCSAPDT  792
ACAD11          397   AEKEVTEF[ 8]DKWGKPlviDKLKEMAKVEGLWNLFLPAVS-   --GLS-HVDYALIAEETGK--C-Ff-APDVFNCQAPDT  473
ACAD12          172   LEPELQRH[ 4]NRWSPSpliEDLKEKAKAEGLWNLFLPLETD[5]GAGLT-NVEYAHLCEVMGMS-LyASEIFN---CSVPDM  252

LCAD            140   IVMSYITNHGSEEQIKHFIPQMTAGKCIGAIAMTEPG-AGSDLQGIKTNAK--KDGSDWILNGSKVFISNGSLSDVVIVV  216
MtbCNF74574      94   INGGYLRQLCTEEQKRRWFPGYCSGEIITAIAMTEPA-AGSDLQGIKTTAV--KDGDDYVLNGSKTFISNGILADLVIVV  170
IVD             132   LCINQLVRNGNEAQKEKYLPKLISGEYIGALAMSEPN-AGSDVVSMKLKAE--KKGNHYILNGNKFWITNGPDADVLIVY  208
SCAD            122   LYLGPILKFGSKEQKQAWVTPFTSGDKIGCFALSEPG-NGSDAGAASTTAR--AEGDSWVLNGTKAWITNAWEASAAVVF  198
IBD             128   MCAWMIDSFGNEEQRHKFCPPLCTMEKFASYCLTEPG-SGSDAASLLTSAK--KQGDHYILNGSKAFISGAGESDIYVVM  204
MCAD            132   LGQMPIIIAGNDQQKKKYLGRMTEEPLMCAYCVTEPG-AGSDVAGIKTKAE--KKGDEYIINGQKMWITNGGKANWYFLL  208
ACAD9           148   IGLKGIILAGTEEQKAKYLPKLASGEHIAAFCLTEPA-SGSDAASIRSRATlsEDKKHYILNGSKVWITNGGLANIFTVF  226
VLCAD           184   IGFKGILLFCTKAQKEKYLPKLASGETVAAFCLTEPS-SGSDAASIRTSAVpsPCGKYYTLNGSKLWISNGGLADIFTVF  262
Mab4HR3_A       116   GNMEVLHRYGSQEQKEVWLEPLLEGDIRSAFCMTEPDvASSDATNMAATAV--VEGDEVVINGRKWWSTGVGHPDCKVII  193
MtbFaDE26        97   SVAPTIMAYGTDEQKRFFLPRIAAGDLHFSIGYSEPG-AGTDLANLRTTAV--RDGDDYVVNGQKMWTSLIQYADYVWLA  173
MtbFaDE29        93   TVGPTLQAHGSELQKKKFLPAILAGEAHFAIGYTEPE-AGTDLASLRTTAV--RDGDHYIVNGQKVFTTGAHDADYIWLA  169
ACAD10          793   GNMELLVRYGTEAQKARWLIPLLEGKARSCFAMTEPQvASSDATNIEASIR--EEDSFYVINGHKWWITGILDPRCQLCV  870
ACAD11          474   GNMEVLHLYGSEEQKKQWLEPLLQGNITSCFCMTEPDvASSDATNIECSIQ--RDEDSYVINGKKWWSSGAGNPKCKIAI  551
ACAD12          253   GTMEILVRYGTEEQKARWLVPLLEGRIHSCFAMTDRKvASSDASNIEASIK--EEDNSYVINGHKWWTSGILHPHCKLCV  330
```

```
LCAD          217  AVT---NH-EAPSPAHGISLFLVENGMKGFIKGRKLHKMGLKAQ---DTAELFFEDIRLPASALLGEENKGFYYIMKELP  289
MtbCNF74574   171  AKT---DP-SAG--AKGVSLLAVERGMDGFERGRNLDKVGMHAQ---DTAELFFDNVRVPKANLLGEEGMGFIYLMTNLA  241
IVD           209  AKT---DL-AAVPASRGITAFIVEKGMPGFSTSKKLDKLGMRGS---NTCELIFEDCKIPAANILGHENKGVYVLMSGLD  281
SCAD          199  AST---DR-AL--QNKGISAFLVPMPTPGLTLGKKEDKLGIRGS---STANLIFEDCRIPKDSILGEPGMGFKIAMQTLD  269
IBD           205  CRT---GG-PGP---KGISCIVVEKGTPGLSFGKKEKKVGWNSQ---PTRAVIFEDCAVPVANRIGSEGQGFLIAVRGLN  274
MCAD          209  ARS---DPdPKAPANKAFTGFIVEADTPGIQIGRKELNMGQRCS---DTRGIVFEDVKVPKENVLIGDGAGFKVAMGAFD  282
ACAD9         227  AKTEVvD--SDGSVKDKITAFIVERDFGGVTNGKPEDKLGIRGS---NTCEVHFENTKIPVENILGEVGDGFKVAMNILN  301
VLCAD         263  AKTPVtDP-ATGAVKEKITAFVVERGFGGITHGPPEKKMGIKAS---NTAEVFFDGVRVPSENVLGEVGSGFKVAMHILN  338
Mab4HR3_A     194  FMGLT-DP--NAHRYARHSMVLVPMDTPGITVERMLPTMGFYDE-pgGHGVVSFDNVRLPADAFIAGPGKGFEIAQGRLG  269
MtbFaDE26     174  VRTNPeSS--GAKKHRGISVLIVPTTAEGFSWTPV-HTMAGPDT-----SATYYSDVRVPVANRVGEENAGWKLVTNQLN  245
MtbFaDE29     170  CRT---DP--NAAKHKGISILIVDTKDPGYSWTPIILADGAHHT-----NATYYNDVRVPVDMLVGKENDGWRLITTQLN  239
ACAD10        871  FMGKT-DP--HAPRHRQQSVLLVPMDTPGIKIIRPLTVYGLEDA-pgGHGEVRFEHVRVPKENMVLGPGRGFEIAQGRLG  946
ACAD11        552  VLGRTqNT--SLSRHKQHSMILVPMNTPGVKIIRPLSVFGYTDNfhgGHFEIHFNQVRVPATNLILGEGRGFEISQGRLG  629
ACAD12        331  FMGKT-DP--QAPRHQQQSMLLVPMDSPGITVIRPLSVFGLEDP-pgGFAEVQFKDVRVPKENIILGPGRAFEIAQGRLG  406

LCAD          290  QERLLIADVAISASEFMFEETRNYV-KQRKAFGKTVAHLQTVQHKLAELKTHICVTRAFVDNCLQLHEA-KRLD-SAT-A  365
MtbCNF74574   242  RERLSIGATAMAAAEDAFERTLEYC-RTREAFGRPIGKFQHNRFTLAEMKTELTVARSFTDECILKEGG-GELT-ADE-A  317
IVD           282  LERLVLAGGPLGLMQAVLDHTIPYL-HVREAFGQKIGHFQLMQGKMADMYTRLMACRQYVYNVAKACDE-GHCT-AKD-C  357
SCAD          270  MGRIGIASQALGIAQTALDCAVNYA-ENRMAFGAPLTKLQVIQFKLADMALALESARLLTWRAAMLKDN-KKPF-IKE-A  345
IBD           275  GGRINIASCSLGAAHASVILTRDHL-NVRKQFGEPLASNQYLQFTLADMATRLVAARLMVRNAAVALQE-ERKD-AVALC  351
MCAD          283  KTRPVVAAGAVGLAQRALDEATKYA-LERKTFGKLLVEHQAISFMLAEMAMKVELARMSYQRAAWEVDS-GRRN-TYY-A  358
ACAD9         302  SGRFSMGSVVAGLLKRLIEMTAEYA-CTRKQFNKRLSEFGLIQEKFALMAQKAYVMESMTYLTAGMLDQPGFPD-CSIEA  379
VLCAD         339  NGRFGMAAALAGTMRGIIAKAVDHA-TNRTQFGEKIHNFGLIQEKLARMVMLQYVTESMAYMVSANMDQ-GATD-FQIEA  415
Mab4HR3_A     270  PGRVHHAMRLIGLAEVALEHACRRG-LDRTAFGKPLVNLGGNRERIADARIAINQTRLLVLHAAWLLDTVGIMG-ALSAV  347
MtbFaDE26     246  HERVALVS--PAPIFGCLREVREWAqNTKDAGGTRLIDSEWVQLNLARVHAKAEVLKLINWELASSQSGPKDAGpSPADA  323
MtbFaDE29     240  NERVML--GPAGRFASIYDRVHAWA-SVPGGNGVTPIDHDDVKRALGEIRAIWRINELLNWQVASAGEDINMA-----DA  311
ACAD10        947  PGRIHHCMRLIGFSERALALMKARV-KSRLAFGKPLVEQGTVLADIAQSRVEIEQARLLVLRAAHLMDLAGNKA-AALDI  1024
ACAD11        630  PGRIHHCMRTVGLAERALQIMCERA-TQRIAFKKKLYAHEVVAHWIAESRIAIEKIRLLTLKAAHSMDTLGSAG-AKKEI  707
ACAD12        407  RARIHNSMRLIGYSERALALMKTRV-MSRTAFGKPLVEQGTILADIARSRVEIEQARLLVLKAAHLMDVAGNKA-AALEM  484

LCAD          366  CMAKYWASELQNSVAYDCVQLHGGWGYMWEYP   IAKAYVD   ARVQPIYGGTNEIMKELIAREIVFDK---         430
MtbCNF74574   318  AMLKWWNTELLKRVVDRCVQLHGGYGYMTEYP   IAKAYQD   VRIQTIFGGTTEIMKEIIGRGLGV-----         380
IVD           358  AGVILYSAECATQVALDGIQCFGGNGYINDFP   MGRFLRD   AKLYEIGAGTSEVRRLVIGRAFNADFH--         423
SCAD          346  AMAKLAASEAATAISHQAIQILGGMGYVTEMP   AERHYRD   ARITEIYEGTSEIQRLVIAGHLLRSYRS-         412
IBD           352  SMAKLFATDECFAICNQALQMHGGYGYLKDYA   VQQYVRD   SRVHQILEGSNEVMRILISRSLLQE----         415
MCAD          359  SIAKAFAGDIANQLATDAVQILGGNGFNTEYP   VEKLMRD   AKIYQIYEGTSQIQRLIVAREHIDKYKN-         425
ACAD9         380  AMVKVFSSEAAWQCVSEALQILGGLGYTRDYP   YERILRD   TRILLIFEGTNEILRMYIALTGLQHAGRI[174]   621
VLCAD         416  AISKIFGSEAAWKVTDECIQIMGGMGFMKEPG   VERVLRD   LRIFRIFEGTNDILRLFVALQGCMDKGKE[172]   655
Mab4HR3_A     348  SEIKVAAPNMAQQVIDMAIQIHGGGGLSNDFP   LAAAWVN   ARALRLADGPDEVHRGVVARIELAKYAND         415
MtbFaDE26     324  SAAKVFGTELATEAYRLLMEVLGTAATLRQNS[8]VERMHRA   CLILTFGGGTNEVQRDIIGMVALGLPRAN[  1]   400
MtbFaDE29     312  AATKVFGTERVQRAGRLAEEIVGKYGNPAEPD   TAELLRW[7]NLVITFGGGVNEVMREMIAASGLKVPRVP[  1]   387
ACAD10       1025  AMIKMVAPSMASRVIDRAIQAFGAAGLSSDYP   LAQFFTW   ARALRFADGPDEVHRATVAKLELKHRI--         1090
ACAD11        708  AMIKVAAPRAVSKIVDWAIQVCGGAGVSQDYP   LANMYAI   TRVLRLADGPDEVHLSAIATMELRDQAKR[  5]   780
ACAD12        485  AMIKMVVPSMANQVIDRAIQAFGAAGLSSDYP   LAQFFGW   AQTLRLGD-----DQLKVAKMELKNQSRL[  8]   555
```

Fig. S3

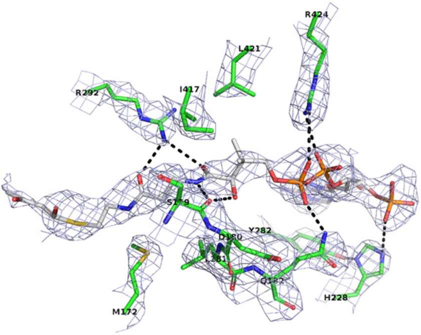
R424
L421
R292
I417
S179
Y282
D180
L181
Q182
H228
M172
R424
L421
R292
I417
S179
Y282
D180
H228
M172

Fig. S4

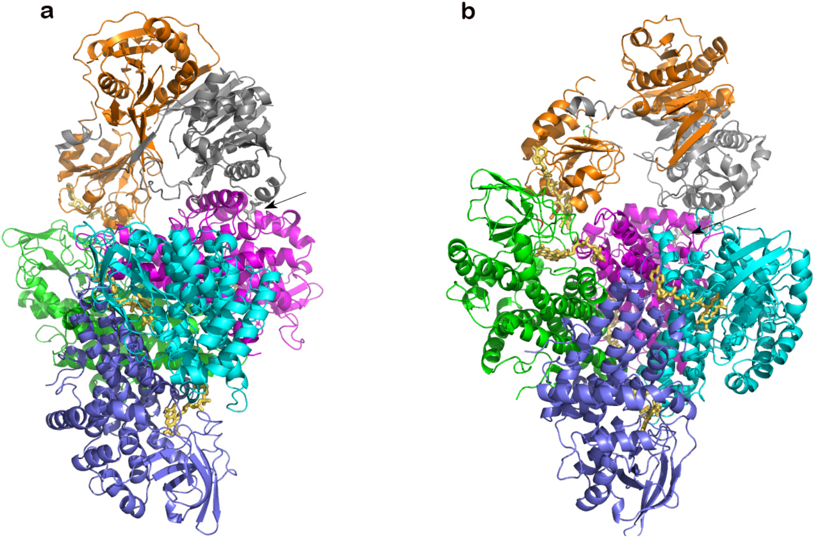

Supplement: Supplementary file 1 — Supplementary Figures. [file 41598_2024_63027_MOESM1_ESM.pdf]
